# Metagenomes from cyanobacterial harmful algal blooms from lakes in Ohio (USA)

**DOI:** 10.1128/mra.00400-25

**Published:** 2025-07-07

**Authors:** Forrest W. Lefler, David E. Berthold, Maximiliano Barbosa, Jing Hu, Autumn Taylor, Jéssica Moretto, Justin D. Chaffin, Heather Raymond, H. Dail Laughinghouse

**Affiliations:** 1 Agronomy Department, Fort Lauderdale Research and Education Center, University of Florida, Davie, Florida, USA; 2 F.T. Stone Laboratory and Ohio Sea Grant, The Ohio State University 2647, Put-in-Bay, Ohio, USA; 3 Office of Research and Graduate Education, College of Food, Agricultural, and Environmental Sciences, The Ohio State University 2647, Columbus, Ohio, USA; Montana State University, Bozeman, Montana, USA

**Keywords:** *Microcystis*, *Planktothrix*, *Dolichospermum*, cyanotoxin

## Abstract

We report two metagenomic libraries of freshwater cyanobacterial harmful algal blooms (cyanoHABs) from Ohio, USA. One sample was collected from a *Planktothrix*-dominated bloom in Grand Lake St. Marys, and another from a mixed *Dolichospermum* and *Microcystis*-dominated cyanoHAB in the western basin of Lake Erie.

## ANNOUNCEMENT

Cyanobacterial harmful algal blooms (cyanoHABs) are a global problem (1), causing human health issues and economic impacts. In Ohio, USA, 45% of the lakes are considered hypereutrophic and experience cyanoHABs (2). Grand Lake St. Marys (GLSM) is among the most hypereutrophic lakes in North America (2), with persistent microcystin-producing *Planktothrix agardhii*-dominated cyanoHABs, and Lake Erie (LE) frequently experiences *Microcystis*-dominated cyanoHABs in the western basin (3, 4). To better characterize these cyanoHABs, cyanoHABs-dominated water samples were collected from GLSM (40.5427–84.5713; subsurface) during March 2021 and from LE (41.6883–82.7931; concentrated 64 µm plankton net) during September 2022. At the lab, samples were filtered in triplicate on 0.22 µm mixed cellulose filters (MilliporeSigma, Burlington, MA, USA) until clogging, immediately flash-frozen in liquid nitrogen, and stored at −80°C.

DNA was extracted using QIAGEN DNeasy Blood & Tissue kit (Qiagen, Hilden, Germany) with modified protocols established by Djurhuus et al. (5). DNA quality was assessed via Nanodrop (Thermo Fisher Scientific, Waltham, MA) and quantity via QUBIT 4.0 dsDNA BR assay (Invitrogen, Waltham, MA). Libraries were prepared using the Nextera mate pair library preparation kit (Illumina, San Diego, CA) following manufacturer’s protocols. Sequencing was conducted using paired-end 2 × 150 bp Illumina NovaSeq 6000 (Novogene, Beijing, China). A total of 53 and 223.6 million paired-end reads were generated from GLSM and LE, respectively. Adaptor sequences and low-quality reads were removed using Fastp 0.24.0 (6) with the option “-D -g -l 150” to remove duplicates, polyG tails, and reads <150 bp, respectively. Reads mapping to *Homo sapiens* and Phi X 174 genomes were removed using BowTie2 v.2.5.4 (7). Reads were quality checked using FastQC v.0.11.9 (8). After quality control, 36 and 184 million paired-end reads remained from GLSM and LE, respectively.

Contigs were *de novo* assembled using metaSPAdes v.4.0.0 (9). Viral contigs were identified and taxonomically classified with geNomad v.1.8.1 (10), and viral metagenome-assembled genomes (MAGs) with direct terminal repeats were quality checked with CheckV v.1.0.1 (11). The remaining contigs were classified as eukaryotic or prokaryotic using Whokaryote v.1.1.2 (12). Prokaryotic contigs were then binned using MetaBAT2 v.2.17 (13), SemiBin2 v.2.1.0 (14), COMEbin v.1.0.3 (15), MetaCoAG v.1.2.2 (16), and MetaDecoder v.1.0.19 (17); bins from all programs were then refined using Binette v.1.0.5 (18). Final bin, now MAG, quality was assessed using CheckM2 v.1.0.1 (19), and taxonomy was assigned using GTDB-tk (R220) v.2.4.0 (20). Medium- (≥50% completion, <10% contamination) to high-quality (>90% completion, <5% contamination) MAGs, as defined by minimum information about a MAG (MIMAG) standards (21), were kept; information on the cyanobacterial can be found in Table 1. Default parameters were used for all programs, unless specified. Viral MAGs were annotated using MetaCerberus v.1.4.0 (22) using KEGG (23), COG (24), CAZy (25), PFAM (26), TIGRfams (27), PGAPfams (28), AMRFinder-fams (29), VOGDB (30), pVOG (31), PHROG (32), and NFixDB (33). Bacterial MAGs were annotated with PGAP (28).

**TABLE 1:** Information of the cyanobacterial MAGs

MAG	Taxonomy	Completeness	Contamination	Genome size (bp)	GC%	CDS	Contigs	N50	GenBank assembly accession
GLSM_49081	Bacteria; Cyanobacteriota; Cyanobacteriia; Cyanobacteriales; Microcoleaceae; *Planktothrix*; *Planktothrix agardhii*	99.74	0.00	4,155,328	40	3,816	395	16,995	GCA_049243445.1
GLSM_23755	Bacteria; Cyanobacteriota; Cyanobacteriia; Gloeomargaritales	51.46	1.04	353144	33	545	115	4,717	GCA_049243845.1
LE_492697	Bacteria; Cyanobacteriota; Cyanobacteriia; Cyanobacteriales; Microcoleaceae; *Limnoraphis*; *Limnoraphis robusta*_A	97.98	0.28	6,442,707	42	5,625	680	16,042	GCA_049240385.1
LE_457126	Bacteria; Cyanobacteriota; Cyanobacteriia; Cyanobacteriales; Microcoleaceae; *Planktothrix*; *Planktothrix agardhii*	99.48	0.00	4,268,989	40	3,894	413	18,238	GCA_049240745.1
LE_379153	Bacteria; Cyanobacteriota; Cyanobacteriia; Cyanobacteriales; Microcystaceae; *Microcystis*; *Microcystis aeruginosa*	88.56	9.92	4,081,143	44	4,082	1,368	3,634	GCA_049241145.1
LE_401666	Bacteria; Cyanobacteriota; Cyanobacteriia; Cyanobacteriales; Nostocaceae; *Dolichospermum*	51.34	1.80	5,530,621	37	4,792	1,789	3,933	GCA_049240865.1
LE_495048	Bacteria; Cyanobacteriota; Cyanobacteriia; Cyanobacteriales; Nostocaceae; *Dolichospermum*; *Dolichospermum* sp001672075	99.63	0.31	4,732,023	37	4,555	405	23,451	GCA_049240365.1
LE_183617	Bacteria; Cyanobacteriota; Cyanobacteriia; PCC-6307	53.30	2.54	114,791	27	135	7	34,328	GCA_049242905.1
LE_186911	Bacteria; Cyanobacteriota; Cyanobacteriia; PCC-6307; Cyanobiaceae; CBW1002	78.03	0.56	2,680,599	69	2,926	250	17,573	GCA_049242845.1
LE_553605	Bacteria; Cyanobacteriota; Cyanobacteriia; PCC-6307; Cyanobiaceae; *Cyanobium*	59.51	3.44	1,908,029	64	2,684	838	2,616	GCA_049239985.1
LE_491439	Bacteria; Cyanobacteriota; Cyanobacteriia; Pseudanabaenales; Pseudanabaenaceae; *Pseudanabaena*; *Pseudanabaena* sp020404895	95.69	0.93	4,395,979	42	4,677	763	8,336	GCA_049240465.1

We confirmed *Planktothrix agardhii* as the dominant cyanobacterium in the GLSM sample, whereas the LE sample had a mixed community of *Limnoraphis robusta, Planktothrix agardhii, Microcystis aeruginosa, Dolichospermum* spp.*, Cyanobium* sp., and *Pseudanabaena* sp.

## Data Availability

All data are deposited at the NCBI under BioProject PRJNA1232823. The SRA accession numbers for the quality control reads are SRR32601175 and SRR32601174 for GLSM and Lake Erie, respectively. The BioSample accession numbers for the assembled metagenomic contigs are SAMN47248468 and SAMN47248469 for GLSM and Lake Erie, respectively. The metagenome-assembled genomes are deposited in NCBI, and their information and accession numbers can be found at https://zenodo.org/records/15733741. Scripts for analyses can be found at https://github.com/flefler/Ohio_Metagenome.
